# Case of Extensive Injury of the Head

**Published:** 1862-07

**Authors:** H. Wanzer

**Affiliations:** Chicago


ARTICLE XXII.

### CASE OF EXTENSIVE INJURY OF THE HEAD.

By H. WANZER, M.D., of Chicago.

I was summoned to Mrs. Murphy, by officer Ensworth, on the night of the 14th May, at a late hour of the night. Found her on Chicago Avenue, lying upon the ground, in front of the house of Mr. Morrison, upon her face. Her head, face, and dress was completely covered with blood, so that I could not tell what color she was. Her extremities and her surface were cold, showing that the blood had left the surface. The pulse was nearly imperceptible at the wrist. The loss of blood, together with the shock she had received by the injuries, had nearly induced a fatal prostration.' She was, in a word, in a state of collapse. I ordered her to be taken into the house of Mr. Morrison; and there commenced my examination for injuries, commencing at the head, I found a transverse cut over the temporal bone, about four inches. The structures were divided to the bone. The second was a transverse cut over the parietal bone, the structures being divided completely to the bone. The third and forth cuts were over the occipital bone; one was perpendicular, the other transverse, about four inches each, dividing the structures completely to the bone, ripping up occipito.frontælis muscle from the bone, for the space of three and a-half inches, making rather an unsightly wound, in the form of a letter T. The perpendicular cut was a bad one, the instrument had penetrated the outer table and diploe of the skull, gouging it out as it were, so that I passed my index-finger freely over the inner table of the skull, the whole length of the cut, only that structure intervening between it and the brain. After cleansing the wounds from hair, and all foreign substances, I brought the parts in apposition by interrupted sutures, adhesive straps, and bandages.

Having finished about the head, I commenced looking for other injuries, and my attention was called to the right shoulder. I found it dislocated, the head of the humerus was downwards, resting upon the axillary plexus of nerves, and manipulating the arm caused her intense suffering. The shoulder, and arm its whole length, were intensely swollen; to the ends of the fingers the blood had infiltrated and extravasated through nearly the entire extent, and upon moving the arm, I heard distinct crepitus of bone, indicating that there was fracture in the neighborhood; and finding none at the arm or hand, I proceeded, assisted by officer Ensworth, to reduce the dislocation at the shoulder. I commenced by extension, which much relieved her, taking the pressure off those plexus of nerves. The dislocation was a bad one, and it was reduced with difficulty; the head of the bone went into its old place with an audible snap, which relieved her at once. I then commenced further examination for the fracture, and found that it was the right scapula or shoulder-blade that was broken. I then placed a pad in the arm-pit, and adjusted a figure of 8 bandage, which met both indications of fracture and dislocation, and pursued my examinations for other injuries; found some of her front teeth knocked out, probably by the same deadly instrument. I also found two old fractures and cicatrices, done, she told me, by the same hands—her husband's. The first had been a complete fracture of the radius and ulna of the other arm; badly set and much deformed. The other was a fracture of the nose. The injuries she has received to her arms will impair her usefulness for obtaining herself and little ones a livelihood, if she ever recovers from them. Having done all I could for the poor woman, I left her for the night, leaving directions to have her carried home, expecting on my next visit to find her dead; but in the morning reaction was well established. The machinery of life, suspended for awhile, had commenced moving, and she has been convalescing ever since. She is a temperate woman, and of a good plastic diathesis. Her cuts about the head nearly all healed by the first intention, excepting the last. She is doing well.

I remarked that she was a temperate woman. Alcoholic drinks of all kinds retard the healing process. They change the essential properties of the blood, by diminishing its plasticity. They promote the suppurative diathesis. Wounds heal slowly and with difficulty after injuries or cutting operations, in those who habitually use them.

